# Genome Wide Association Mapping in *Arabidopsis thaliana* Identifies Novel Genes Involved in Linking Allyl Glucosinolate to Altered Biomass and Defense

**DOI:** 10.3389/fpls.2016.01010

**Published:** 2016-07-12

**Authors:** Marta Francisco, Bindu Joseph, Hart Caligagan, Baohua Li, Jason A. Corwin, Catherine Lin, Rachel E. Kerwin, Meike Burow, Daniel J. Kliebenstein

**Affiliations:** ^1^Department of Plant Sciences, University of California, DavisDavis, CA, USA; ^2^Group of Genetics, Breeding and Biochemistry of Brassicas, Department of Plant Genetics, Misión Biológica de Galicia, Spanish Council for Scientific ResearchPontevedra, Spain; ^3^DynaMo Center, University of CopenhagenCopenhagen, Denmark

**Keywords:** Arabidopsis, allyl GSL, plant biomass, defense metabolism, GWAS, novel genes

## Abstract

A key limitation in modern biology is the ability to rapidly identify genes underlying newly identified complex phenotypes. Genome wide association studies (GWAS) have become an increasingly important approach for dissecting natural variation by associating phenotypes with genotypes at a genome wide level. Recent work is showing that the *Arabidopsis thaliana* defense metabolite, allyl glucosinolate (GSL), may provide direct feedback regulation, linking defense metabolism outputs to the growth, and defense responses of the plant. However, there is still a need to identify genes that underlie this process. To start developing a deeper understanding of the mechanism(s) that modulate the ability of exogenous allyl GSL to alter growth and defense, we measured changes in plant biomass and defense metabolites in a collection of natural 96 *A. thaliana* accessions fed with 50 μM of allyl GSL. Exogenous allyl GSL was introduced exclusively to the roots and the compound transported to the leaf leading to a wide range of heritable effects upon plant biomass and endogenous GSL accumulation. Using natural variation we conducted GWAS to identify a number of new genes which potentially control allyl responses in various plant processes. This is one of the first instances in which this approach has been successfully utilized to begin dissecting a novel phenotype to the underlying molecular/polygenic basis.

## Introduction

Exposure of plants to biotic and abiotic stress induces a disruption in plant metabolism implying physiological costs, and thus leading to a reduction in fitness and ultimately in productivity (Karban and Baldwin, 1997; Baldwin, 1998; Mauricio, 1998; Cipollini et al., 2003; Paul-Victor et al., 2010; Züst et al., 2011). In the course of evolution, plants have evolved a myriad of defense mechanisms, which include enhanced production of secondary metabolites such as phenolics, terpenoids, alkaloids, and glucosinolates (GSL) allowing them to adapt and survive stressful events (Ramakrishna and Ravishankar, 2011; War et al., 2012). GSLs are sulfur-containing secondary metabolites that are the most important groups of *Brassicaceae* metabolites derived from amino acid biosynthesis. Like other secondary metabolites, GSL are not directly involved in providing energy or structural components key for a plant's growth and development but they are essential for the plants survival through ecological interactions with the environment. GSLs are key factors controlling plant resistance against a broad suite of biotic attackers in Arabidopsis and other Brassicas (Lambrix et al., 2001; Kliebenstein et al., 2002; Beekwilder et al., 2008; Hansen et al., 2008; Fan et al., 2011; Bednarek, 2012).

Over the past decades, research has identified nearly the complete catalog of genes and enzymatic steps within the GSL biosynthetic pathways, including the identification of transcription factors (TFs) regulating the aliphatic GSL pathway, allowing for detailed studies on synthesis and regulation of these compounds (Wittstock and Halkier, 2002; Grubb and Abel, 2006; Halkier and Gershenzon, 2006; Gigolashvili et al., 2007, 2008; Hirai et al., 2007; Sønderby et al., 2007, 2010; Schweizer et al., 2013). From these studies, a model was developed that followed the standard hierarchical regulatory architecture for plant defenses, in which biotic attackers are perceived and signals transmitted via the JA-ILE controlled MYC2/3/4 TFs to modulate the expression of the aliphatic GSL pathway in conjunction with the MYB28/29/76 TFs (Gigolashvili et al., 2007, 2008; Hirai et al., 2007; Sønderby et al., 2007, 2010; Schweizer et al., 2013). Recently, this model has been expanded to include a wider array of TFs that interact with the pathway, showing that signal integration can occur at the promoter level of the pathway and not solely rely on integration prior to JA-ILE (Li et al., 2014). Thus, there is a complex suite of external stimuli that can modulate the expression of the pathway.

Challenging the hierarchical model where the defense is solely an output of a regulatory network is new evidence suggesting that GSL metabolites and genes can have a regulatory influence on itself and other pathways. The accumulation of specific GSL have been shown to impact plant growth in a manner that was occasionally considered to be solely caused by the metabolic cost of the GSLs production (Delarue et al., 1998; Barlier et al., 2000; Hansen et al., 2001; Chen et al., 2003; Mikkelsen et al., 2004). However, more recent work suggests that these changes in growth linked to GSL accumulation are more likely the consequence of regulatory cross-talk between the GSL pathway and hormone metabolism (Züst et al., 2011). The introduction of a functional AOP2, a biosynthetic enzyme in the aliphatic GSL pathway, into a naturally occurring knockout genotype leads to alterations in flowering, JA-ILE mediated defense signaling and oscillatory behavior of the circadian clock (Wentzell et al., 2007; Kerwin et al., 2011; Burow et al., 2015). While these papers suggested that at least some of these effects are caused by the AOP2 RNA, additional research showed that a GSL metabolite produced by AOP2 enzyme, allyl GSL (also known as 2-propenyl GSL or sinigrin), function as a signal that alters plant biomass and metabolism in Arabidopsis (Francisco et al., 2016). Similarly, studies have shown that a product resulting from the indole GSL pathway can regulate the production of callose in response to pathogen attack (Bednarek et al., 2009; Clay et al., 2009). These studies showed that a specific GSL metabolite may provide potential regulatory information to influence the general behavior of the plant. However, there is still a need to test how ally GSL modulates these processes and to identify the genes and mechanisms that may facilitate this.

Effective developing methodology to elucidate genes underlying complex traits is the use of natural variation through genome wide association mapping studies (GWAS). GWAS combines phenotype and single-nucleotide polymorphism (SNP) data from natural populations to study the genetic basis of heritable phenotypes, providing valuable information for gene hunting, understanding of biological processes, and plant breeding (Borevitz et al., 2007; Atwell et al., 2010; Chan et al., 2010a, 2011; Brachi et al., 2015; Corwin et al., 2016). The most extensive use of GWAS in Arabidopsis has been testing of well-studied traits such as flowering time variation or disease resistance (phenotypes controlled by single genes with very large effects) where strong phenotype-SNP associations have been found for candidate genes identified *a priori* from molecular genetic studies (Atwell et al., 2010). The use of GWAS with previously studied polygenic traits allowed the identification of both previously known genes and numerable validatable candidate genes (Borevitz et al., 2007; Chan et al., 2011; Filiault and Maloof, 2012; Corwin et al., 2016). While this suggests that GWAS may be an efficient way to uncover candidate genes for novel phenotypes that have no previous mechanistic information, there is relatively little precedent for this use of GWAS.

To start dissecting the mechanisms behind exogenous allyl GSL induced responses we measured changes in plant biomass and defense metabolites in a collection of natural 96 *Arabidopsis thaliana* accessions fed with 50 μM of allyl GSL. Exogenous GSL was introduced exclusively to the roots and the compound was transported up to the leaf and caused a wide range of heritable effects upon plant biomass and endogenous GSL accumulation. Using natural variation we conducted GWAS to identify and validate a number of new genes which potentially control allyl signaling feedback inhibition. This identified eight genes that can influence the link between allyl GSL and either biomass accumulation or defense chemistry. These genes include known genes in different pathways and completely unstudied genes. This is one of the first instances in which this approach has been successfully utilized to dissect a novel phenotype to the underlying molecular/polygenic basis.

## Material and methods

### Plant material and exogenous allyl GSL feeding experiment

A set of 96 natural *A. thaliana* accessions was analyzed (Nordborg et al., 2002, 2005; Borevitz et al., 2007; Atwell et al., 2010; Chan et al., 2010a,b, 2011; Francisco et al., 2016). Seeds were surface-sterilized (1-min, 70% ethanol soaking followed by a 20-min, 50% sodium hypochlorite), rinsed (five times) in sterile, distilled water. They were then placed on petri dishes containing half-strength Murashige and Skoog (MS) salt medium (CAISSON, MSP01-1LT) adjusted to pH 5.8, containing 0.8% agar, and 1% sucrose concentration (control). To study the effect of exogenous allyl GSL on plant biomass and metabolite content, 0.22 μm filter sterilized allyl GSL 100 mM stock solution (Sigma S1647-1G) was added to the autoclaved MS (at 55⋅C) to a final concentration of 50 μM (treatment). Seeds were placed in 36 grid square 100 × 15 mm plates with 50 mL of medium. Five plants per accession were grown in a randomized partial block design (one seed per grid square). Seeds were planted on control (MS) and allyl-containing MS (MS + Allyl) to provide five measurements per accession per treatment. After planting on media, plates were stratified for 3 days in the dark at 4⋅C to break dormancy. Plates were then transferred to a growth chamber under long-day conditions (16 h light at 100–120 μEi, 20⋅C). Any seedlings with leaf contact to the agar were removed from the analysis to ensure that root-to-shoot transport had occurred. At 15 days post germination, the rosette of each seedling was harvested from the plates, weighed to record the plant fresh weight (fw), then placed into a 96-deep well tube containing 90% methanol for GSL extraction and analyzed for GSL content as described below.

### Analysis of GSL content

GSL of excised shoots were measured using a previously described high-throughput analytical system (Kliebenstein et al., 2001a, b, c). Briefly, rosettes of all seedlings were individually removed from plates with forceps, weighed and placed in a single well of 96-well microliter plate containing 400 μL of 90% methanol and one 3.8 mm stainless steel ball-bearing. Tissues were homogenized for 3 min in a paint shaker, centrifuged, and the supernatants transferred to a 96-well filter plate with 50 μL of DEAE sephadex and washed once with water. The sephadex-bound GSL were eluted by overnight, room temperature incubation with 110 μL of sulfatase. Individual desulfo-GSL within each sample was separated and detected by HPLC-DAD, identified, and quantified by comparison to purified standards. The GSL traits are reported as μmol g of fw of each plant. All seedlings were measured individually and GSL abundance was normalized to the fresh weight. In addition to the content of individual GSL, we developed a series of summation and ratio traits based on prior knowledge of the GSL pathways (Table S1; Kliebenstein, 2007; Wentzell et al., 2007).

### Genome wide association mapping

We obtained the least-square means for plant biomass and all GSL traits in all the accessions for both the treated and untreated seedlings. We used these values for GWA using a ridge regression approach that models all polymorphisms in a single model as random effects to predict the *H*^2^ model-corrected genotypic accessions means for each phenotype (Shen et al., 2013). From this model, the heteroscedastic effects (HEM) were extracted for each polymorphism. Since determining the degrees of freedom for random variables is difficult, a significant effect threshold was estimated by permuting the phenotypic means across the accession backgrounds 1000 times and taking the 99th quantile. Individual genes were considered associated with the phenotype if they had at least 2 significant SNPs in their coding region, similar to the method used in Chan et al. (2011). For the GSL traits, we focused only on the traits that were measurable in all accessions to maximize the information from the accessions and to minimize the signal from the natural variation controlling the GSL profile.

### Single gene validation

To validate the ability of specific genes to influence the response to exogenous allyl application, we measured biomass and GSL content of 17 T-DNA insertion lines from 13 candidate genes (Table 1; Haughn et al., 1991; Kliebenstein et al., 2001a; Hansen et al., 2007; Sønderby et al., 2007, 2010; Li et al., 2008). For these analyses, 10 plants per T-DNA line were grown in a randomized partial block design and the entire experiment was performed four times using the same design providing 40 measurements per genotype per treatment.

**Table 1:** **Description of the T-DNA insertion lines on candidate genes selected from GWAS study**.

**Gene ATG #**	**T-DNA line**	**Name**
AT3G01970	GABI_684G12	WRKY DNA-binding protein 45
AT3G16770	SALK_030459C	Ethylene-responsive element binding protein (ERF72)
AT5G45950	SALK_082692C	GDSL-like Lipase/Acylhydrolase superfamily protein
AT1G05680	SALK_001830C	Uridine diphosphate glycosyltransferase (UGT74E2)
	SALK_091130C	
	SALK_016116C	
AT1G69490	SALK_019747C	NAC-like, activated by AP3/PI (ANAC029)
	SALK_005010C	
AT2G45360	SALK_051976C	Protein of unknown function (DUF1442)
AT3G17520	SALK_099278C	Late embryogenesis abundant protein (LEA) family protein
AT3G24460	SALK_011594C	Serinc-domain containing serine and sphingolipid biosynthesis protein
AT5G67370	SALK_029971C	Protein of unknown function (DUF1230)
AT1G18710	SALK_123009C	Myb domain protein 47 (MYB47)
AT4G16780	SALK_106790C	Homeobox-leucine zipper protein 2 (AtHB2)
	SALK_006502	
AT2G44910	SALK_121097C	Homeobox-leucine zipper protein 4 (AtHB4)
AT3G03040	SALK_151533	F-box/RNI-like superfamily protein

### Statistical analyses

All the relative differences for each trait were calculated as: $\frac{\left(\left(MS+Allyl\right)-\left(\text{MS}\right)\right)}{\left(1∕2\left[\left(\text{MS}+\text{Allyl}\right)+\left(\text{MS}\right)\right]\right)}$[MS stands for MS media with (MS + allyl) or without (MS) exogenous allyl]. To test how the plant biomass and GSL responses to allyl treatment interact with natural variation, the ANOVA utilized accession and treatment (MS and MS + Allyl) as factors and experiment as a random variable. Plate was tested for significance as a random effect in a mixed model but not found to significantly alter the results and hence dropped from the model. The least-square means of each plant biomass and GSL phenotype per each accession within each treatment were obtained using this model. Multiple comparisons were made *post-hoc* using Tukey's *t*-test with P ≤ 0.05 within the model. Nested ANOVA was also utilized to test for the effect of exogenous allyl GSL on plant biomass and GSL content of different T-DNA insertion lines. Each mutant was tested in an individual ANOVA against the Col-0 (WT) genotype. We calculated estimates of broad-sense heritability (H) for plant biomass and allyl accumulation as *H*^2^ = σ${}_{\text{g}}^{2}$/σ${}_{\text{p}}^{2}$, where σ${}_{\text{g}}^{2}$ was the estimated trait genetic variance among different genotypes in this mapping population of 96 Arabidopsis accessions and σ${}_{\text{p}}^{2}$ was the total phenotypic variance for a trait. All statistical analyses were conducted using SAS.

## Results

### Natural variation in arabidopsis biomass responses to allyl GSL

**Figure 1:**
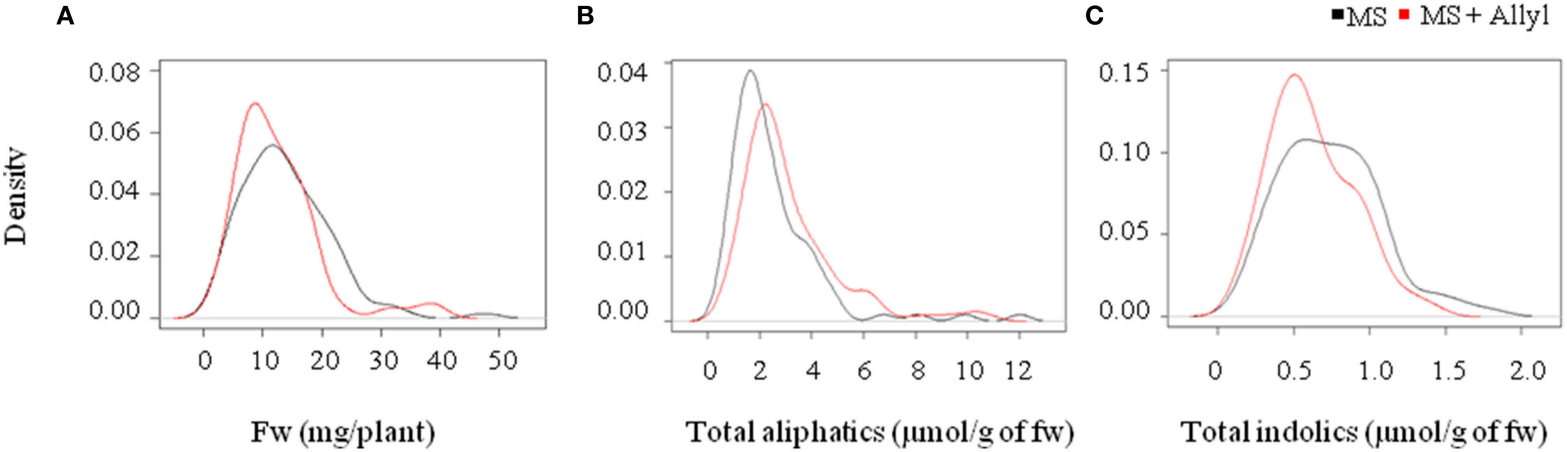
**Natural variation in Arabidopsis biomass and GSL accumulation in response to exogenous allyl GSL. (A)** Kernel density plots showing the distribution of fw (mg/plant), **(B)** total aliphatic GSLs, and **(C)** total indolic GSLs (μmol/g of fw) from 96 natural Arabidopsis accessions grown in MS (black line) and MS + Allyl (red line).

To begin identifying genes and the potential mechanism(s) by which allyl GSL can affect biomass changes in Arabidopsis, we measured the response of a population of 96 natural Arabidopsis accessions to external allyl GSL application. All accessions were planted in quintuplicate using a random split-block design. This population was previously analyzed to assess how endogenous GSL genetic variation influences the link between allyl GSL and biomass accumulation but the genetic architecture of these traits have not yet been described (Francisco et al., 2016). ANOVA showed that plant biomass was highly heritable (*H*^2^ = 0.88) and that natural Arabidopsis accessions have significant variation for the effect of allyl GSL upon seedling plant biomass (Table 2). The distribution of plant biomass across the accessions showed that, in general, exogenous allyl GSL application decreased plant biomass across the population but individual accessions showed positive responses (Figure 1). Thus, there is genetic variation for the plant biomass response to exogenous allyl GSL application in *A. thaliana*. Further, the presence of accessions with positive and negative responses to allyl GSL suggests that there is likely more than one mechanism controlling the response.

**Table 2:** **Allyl treatment affects Arabidopsis plant biomass**.

**Source**	**Degrees of freedom**	**Sums of squares**	***F* value**	***P***
Accession	95	0.0290	6.2	< 0.0001
Allyl Treatment	1	0.0005	10.3	0.0014
Accession × Allyl Treat	95	0.0140	2.9	< 0.0001
Block	4	0.0002	1.1	0.3343
Error	731	0.0280		

Analysis of variance testing of the effect of exogenous feeding of 50 μM allyl GSL upon the biomass in comparison to the control across of 96 Arabidopsis natural accessions. Type III Sums-of-squares are presented.

**Figure 2:**
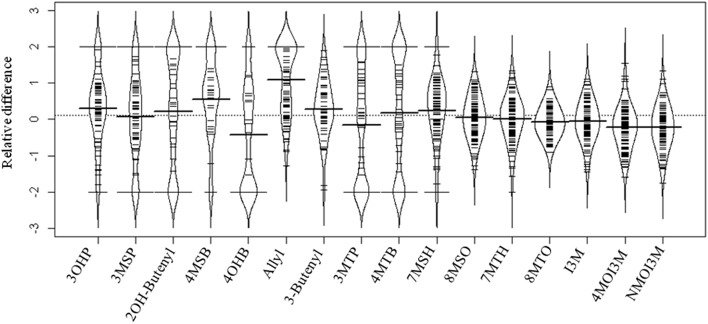
**Distribution of the relative difference of individual GSL accumulation in response to allyl GSL treatment across the Arabidopsis accessions**. A beanplot is used to show the distribution of the change in GSL accumulation between the treated and untreated samples across the accessions using the abbreviations in Table S1. Relative difference was determined as (GSL treatment − GSL control)/(0.5 × [GSL treatment + GSL control]). The dashed line in the middle of the plot is the overall average of the relative GSL difference between control and allyl treatment across all GSL. The thick black line in the middle of each bean for each compound is the mean response for that specific GSL trait across all the accessions. The black colored curved bean pod surrounding the observations is the theoretical probability density distribution of these observations. The small lines represent individual data points, with the length of the line proportional to the number of observations with that specific value. The relative difference between treatment and control varied across the 96 accessions from −2 to 2 for each GSL compound, depending on whether that GSL was present in the treatment compared with control.

To measure the genetic variation in how endogenous GSL accumulation responds to exogenous allyl GSL application, we measured GSL from the 96 Arabidopsis accessions seedlings fed with allyl GSL and from the control samples (Francisco et al., 2016). These were from the same individual seedlings measured for biomass and all values are adjusted to the seedlings' biomass. This analysis detected 14 aliphatic GSL compounds and three indolic GSL compounds from which we could define an additional 15 descriptive variables to isolate specific biosynthetic processes and generate a total of 32 traits (Wentzell et al., 2007; Chan et al., 2010a). The GSL traits significantly varied between the accessions with 29 of the 32 aliphatic and indolic GSL traits showing a significant interaction of accession by allyl GSL treatment, suggesting that the accessions have differential responses to the treatment as measured by GSL accumulation (Table 3; Figure 2). In general, aliphatic GSL accumulation across the accessions tended to increase after the application of allyl GSL (Figure 1). However, like biomass accumulation, these changes in endogenous aliphatic GSL accumulation showed a wide range of both positive and negative responses across the accessions (Figure 2). Further, the positive effects were larger than could be accounted for by the additive effect of exogenous allyl GSL application. Moreover, other GSL that cannot be synthesized from the allyl GSL, such as but-3-enyl GSL were also affected by the allyl treatment showing that this is not caused by the uptake and conversion of allyl GSL to other structures (Figure 2). In contrast to the aliphatic GSL, total indolic GSL content and specific indolic GSL compounds, tended to be reduced in most accessions while a few specific accessions showed an increase (Figures 1, 2). Again, the presence of accessions showing both positive and negative responses suggests that the response to allyl GSL likely involves a number of pathways.

**Table 3:** **Allyl treatment affects Arabidopsis GSL accumulation**.

**GSL**	**Accession**	**Treatment**	**Accession × Treatment**	**Block**
3OHP	19314.4^**^	48.6^*^	2527.0^**^	4.5
3MSP	1531.4^**^	9.7	214.7	15.4
2-OH-butenyl	1286.3^**^	14.3^**^	247.0^**^	1.3
4OHB	609.9	5.4	506.4	31.6
4MSB	5476.7^**^	35.9^**^	535.8^**^	1.8
Allyl	228299.2^**^	2504.4^**^	14655.6^**^	116.4
5MSP	19.9^**^	0.1	4.7^**^	0.2^*^
But-3-enyl	27141.5^**^	245.8^**^	3020.0^**^	25.9
3MTP	304^**^	0.1	128.2^**^	7.2^*^
7MSH	222.3^**^	10.4^**^	114.9^**^	8.8^*^
4MTB	1171.3^**^	5.0^**^	211.8^**^	0.3
8MSO	2271.4^**^	0.2	1058.3^**^	120.0^*^
7MTH	258.4^**^	2.3^*^	78.8^**^	3.8
8MTO	1264.4^**^	4.2	247.0^**^	76.7^*^
I3M	1735.6^**^	21.3	972.8	17.4
4MI3M	132.1^**^	12.2^**^	97.8^**^	2.3
MI3M	1065.9^**^	74.8^**^	397.1^**^	75.0^*^
Total 3C GSL	204277.5^**^	3524.2^**^	18422.4^**^	225.7
Total 4C GSL	73532.8^**^	1128.0^**^	9785.0^**^	88.4
Total 7C GSL	748.6^**^	21.8^**^	301.1^**^	7.1
Total 8C GSL	3903.5^**^	3.8	1491.5^**^	19.5
Short chain GSL	216853.6^**^	8617.8^**^	29799.6^**^	376.3
Long chain GSL	6502.5^**^	7.4	2429.1^**^	48.2
Short vs. Long	2004.5^**^	120.0	389.5^**^	9.8
Total alkyl	288705.0^**^	6012.9^**^	26195.3^**^	134.0
Total MT	3495.1	0.4	911.0^**^	66.9^*^
Total MS	12012.8	166.9^**^	2593.5^**^	364.1^*^
Total aliphatics	267251.2	9432.6^**^	41274.6^**^	579.2
Total indolics	4998.0	281.4^**^	2477.6^**^	193.1^*^
Total GSL	296572.0	6140.8^**^	49237.5^**^	1360.9
Aliphatics vs. indolics	43929.0	763.7^**^	1455.4^**^	92.4^*^
MS/MT ratio	1738.5	41.1^**^	547.2^**^	72.4^**^

Results of the ANOVA for the measured GSL traits from 96 Arabidopsis natural accessions fed with 50 μM of exogenous allyl GSL or grown on control media are shown. The Type III Sums-of-squares or each factor of the model and its significance are listed for each of the GSL traits. A single

* shows that the factor significantly affected the trait (P ≤ 0.05) with

** for P ≤ 0.01. See Table S1 for abbreviations.

### Natural variation in arabidopsis accumulation of exogenous allyl GSL

Approximately half of the studied accessions grown do not produce endogenous allyl GSL because they do not contain a functional copy of the required single-copy gene for the AOP2 enzyme (Kliebenstein et al., 2001a). Thus, any accumulated allyl GSL that we measure within these accessions is solely due to the uptake and transport of exogenous allyl allowing us to test if there are differences in the ability to take up and store this GSL from the growth media. The mean accumulation of allyl GSL was 0.34 μmol/g of fw and ranged from 0.11 to 0.86 μmol/g of fw. A three-way ANOVA identified highly significant differences between accessions for the accumulation of exogenous allyl GSL within the seedling leaves (Table 4). Broad-sense heritability of exogenous allyl GSL accumulation was 70% for the accessions that do not produce endogenous allyl GSL. Thus, there is genetic variation for the ability to import, transport and accumulate the exogenous allyl GSL in the leaves of Arabidopsis seedlings.

**Table 4:** **Analysis of allyl accumulation in foliar tissues of Arabidopsis accessions**.

**Source**	**Degrees of freedom**	**Sums of squares**	***F* value**	***P***
Accession	43	453.7	3.6	< 0.0001
Block	4	6.2	0.5	0.7129
Error	162	469.2		

ANOVA was used to test if the 44 Arabidopsis accessions which do not synthesize endogenous allyl GSL differed significantly for the accumulation of exogenous allyl GSL. Type III Sums-of-squares are presented.

### Genome wide association mapping of biomass and GSL responses to exogenous allyl GSL within arabidopsis accessions

**Figure 3:**
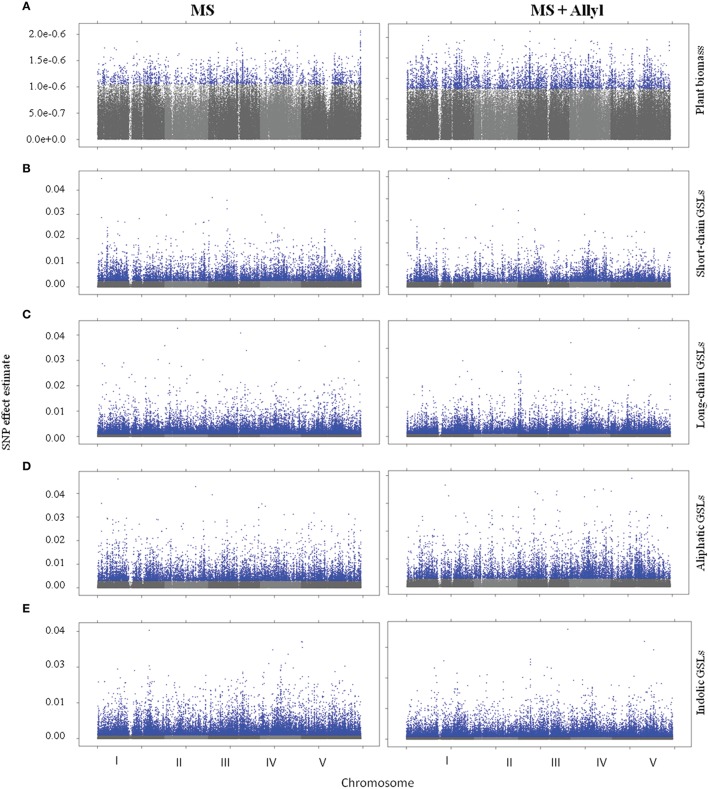
**Manhattan plots of GWAS results**. Genome wide distribution of the absolute value of the heteroscedastic SNP effects. Shades of gray represent nonsignificant SNP effects. Blue points represent significant SNP effects under control (MS) and allyl treatment (MS + Allyl). **(A)** Plant Biomass, **(B)** Short-Chain GSLs, **(C)** Long-Chain GSLs, **(D)** Aliphatic GSLs, **(E)** Indolic GSLs.

To identify genes within Arabidopsis that may control the biomass or GSL accumulation responses to exogenous allyl GSL treatment, we utilized the mean biomass and GSL accumulation in each accession grown with or without allyl GSL to conduct GWA mapping (Figure 3). For these analysis we employed a ridge-regression model that treats all SNPs as random effects (Shen et al., 2013). Using this ridge-regression model we tested all traits for significance associations across 115,301 SNPs with a MAF > 0.2 that covered 19,352 unique genes. Significance thresholds were determined by measuring the 95th percentile of the randomly generated effects of 1000 permutations of the means among the accessions (Chan et al., 2011; Corwin et al., 2016). This permutation threshold, while conservative, allows us to utilize an empirically derived threshold for significance based on the specific phenotypes distribution. We then applied a filter to these SNP lists to find candidate genes by requiring a gene to be considered as a potential GWA candidate only if it has two or more significant SNPs. This approach has previously been shown to identify genes with a high validation success rate for an array of traits (Chan et al., 2011; Corwin et al., 2016). Using this approach we identified 671 genes significantly associated with biomass accumulation with the majority found uniquely in either the control (203) or allyl treated accessions (435) (Tables S2, S3). Only 33 genes were significant GWA candidates using biomass in the presence and absence of allyl GSL. A survey of these genes by either GO analysis or by co-expression network clustering using ATTED-II (Obayashi et al., 2009), did not identify any obvious enrichment patterns.

**Figure 4:**
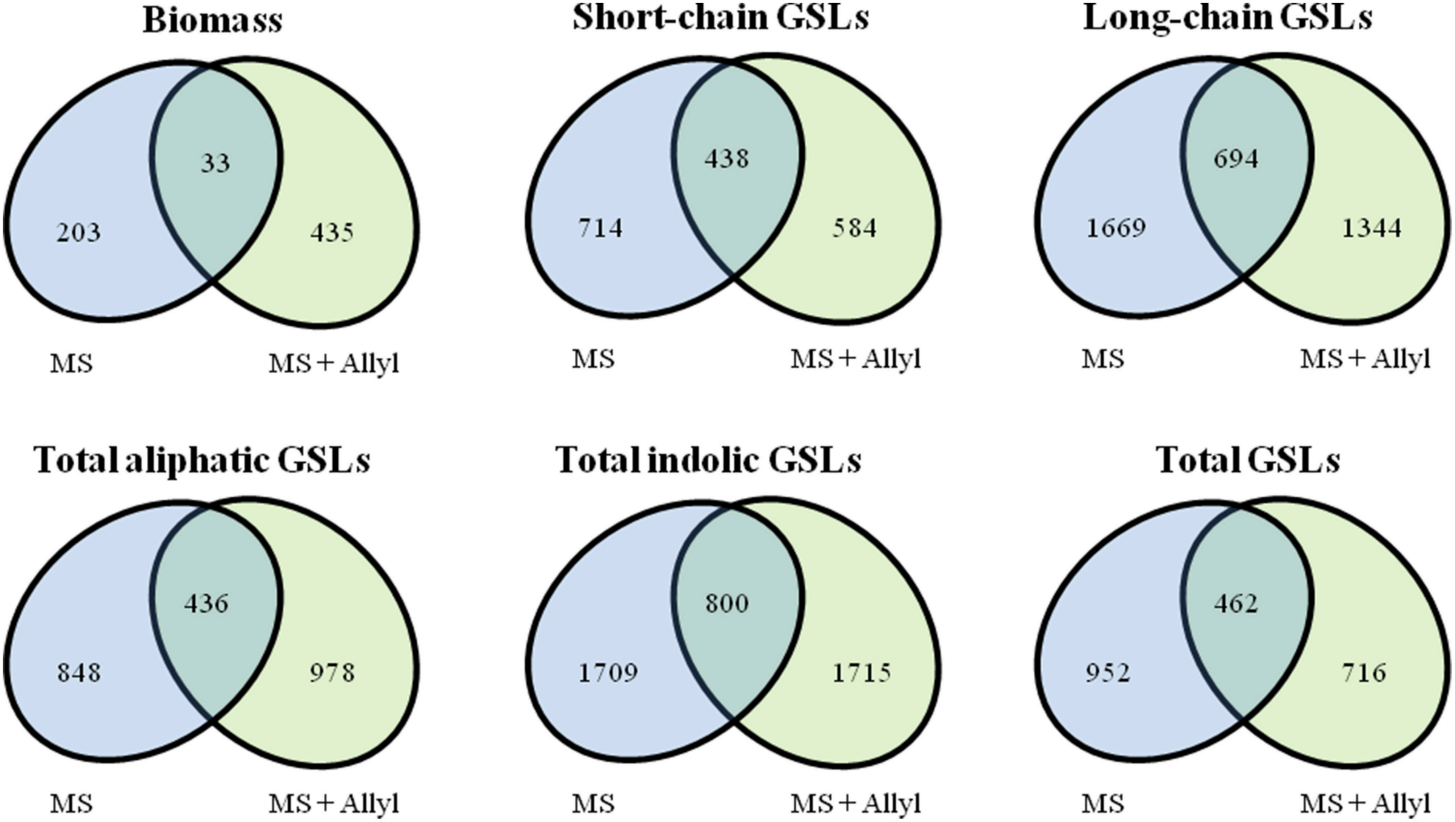
**Overlap of significant GWA candidate genes between control (MS) and treated samples with 50 μM of allyl GSL (MS + Allyl GSL)**. VENN diagram showing common candidate genes identified among the plant biomass, short-chain GSLs, long-chain GSLs, total aliphatic GSLs, total incolic GSLs, and total GSLs traits.

To map genes that influence the variation in endogenous GSL responses to exogenous allyl GSL, we focused on five GSL phenotypes that are present in all accessions, the total amount of long-chain GSLs, total short-chain GSLs, total aliphatic GSLs, total indolic GSLs and the sum of all GSLs (Kliebenstein et al., 2001b,c, 2002; Wentzell et al., 2007; Chan et al., 2010a, 2011). The accumulation of individual GSLs is heavily dependent on presence/absence variation of known GSL enzyme loci leading to presence/absence variation in these compounds and confounds the GWAS mapping. In contrast, these summation based traits are measurable in all accessions and are largely independent of the known major effect GSL polymorphisms as shown by previous GWAS analysis (Kliebenstein et al., 2001b,c, 2002; Wentzell et al., 2007; Chan et al., 2010a, 2011). This increases our power to identify causal genes both by having data for all accessions and by eliminating major effect polymorphisms that can otherwise hinder the power to identify smaller effect loci (Nordborg and Weigel, 2008). Given the quantitative distribution of GSL responses to exogenous allyl treatment, we expected mainly small to moderate effect loci (Figure 3). GWAS with these traits identified on average 2750 genes significantly associated with any given trait (Tables S2, S3). Of these candidate genes, 36% were typically found with the treated samples, 43% with the control treatment and 21% under both conditions (Figure 4). Interestingly, this contrasts with biomass accumulation where only 5% of the genes were found under both conditions. A survey of these genes by either GO analysis or by co-expression network clustering did not identify any obvious mechanistic patterns.

**Figure 5:**
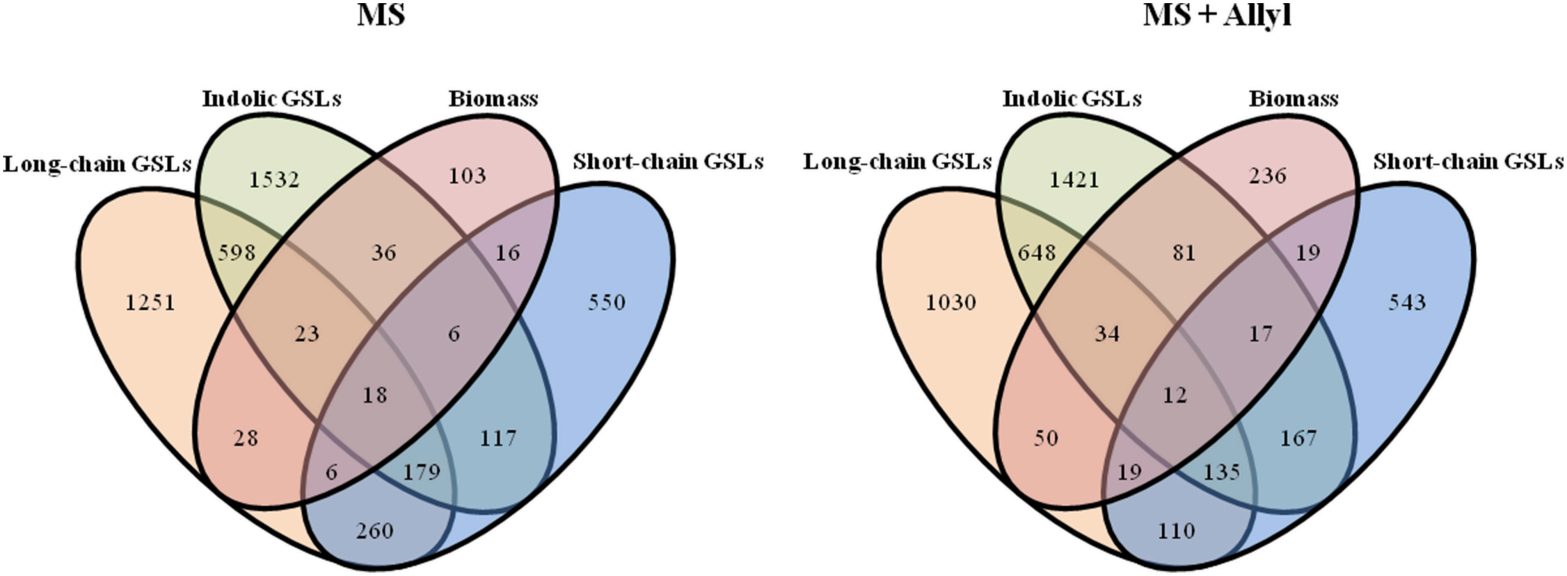
**Overlap of significant GWA candidate genes between plant biomass and GSL phenotypes**. VENN diagram showing common candidate genes identified among the short-chain GSLs, long-chain GSLs, total incolic GSLs and plant biomass traits studied from control (MS) and treated samples with 50 μM of allyl GSL (MS + Allyl).

To check if the GWA mapping identified candidate genes were similar for the biomass and GSL traits, we investigated the overlap of GWA candidate genes identified across plant biomass and three GSL traits that summarize the majority of GSL variation (short-chain GSLs, long-chain GSLs and total indolic GSLs) from control and treated samples (Figure 5). This showed that 133 of the identified GWA candidate gene sets from plant biomass overlap with the identified GWA candidate genes from GSL phenotypes in the control samples. The number of overlap candidate genes between plant biomass and GSL traits was 232 for the exogenous allyl treated samples. Only 27 genes overlapped between biomass and GSL accumulation in both the presence and absence of allyl GSL. This suggests that the effect of the majority of the candidate genes we identified for biomass and GSL phenotypes are conditioned by the exogenous allyl treatment.

### Validation of candidate genes via T-DNA insertion lines

To test if any of the genes identified through GWA mapping may influence the response to exogenous allyl GSL, we filtered the candidate genes that affect plant biomass by removing the genes that were candidates in both conditions. We then further queried the remaining genes to find those whose transcript accumulation is responsive to allyl GSL (Burow et al., 2015). We ranked genes from this list based on fold-change response of their transcripts to allyl GSL. We chose the top 13 most responsive candidate genes and obtained 17 homozygous T-DNA insertion lines (Table 1). This included obtaining two alleles in as many genes as we could given public databases. All of these lines were validated as homozygous and grown concurrently with the wild type (WT) Col-0 in the same growth chamber to obtain seeds to control for maternal environmental effects as much as possible. We then grew all the genotypes in the presence and absence of the exogenous allyl treatment and measured the plant biomass and GSL responses to exogenous allyl GSL. Each genotype within each treatment has a minimum of 40 independent measurements conducted across four experiments using a randomized block design (Table S4).

**Figure 6:**
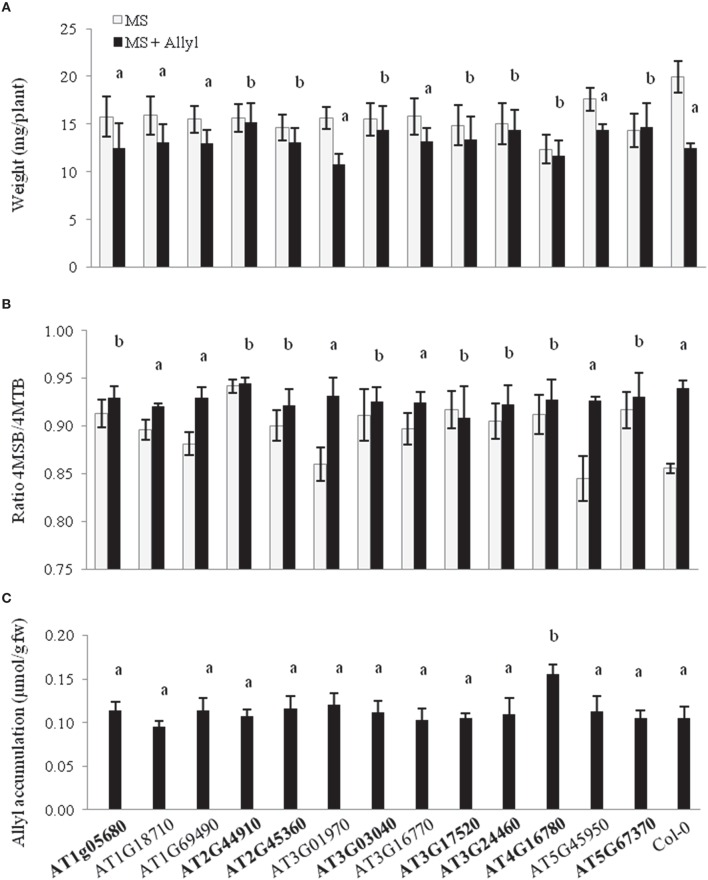
**Plant biomass responses and GSL content variation among T-DNA insertion lines of 13 candidate genes treated with allyl GSL. (A)** Quantification of 15-day-old fw (mg/plant) seedlings from T-DNA insertion lines of 13 candidate genes and wild-type (Col-0) fed with 50 μM of allyl glucosinolate. **(B)** Ratio of 4-methylsulfinylbutyl (4MSB)/4-methylthiobutyl (4MTB) calculated as 4MSB/(4MSB + 4MTB). **(C)** Average allyl GSL accumulation of the evaluated genotypes. The bar chart represents the mean and the standard deviation. Each genotype within each treatment has a minimum of 40 independent measurements conducted across four experiments using a randomized block design. Means with the same letter show if the genotype's response to the treatment was statistically similar to Col-0 (a) or different from Col-0 (b) at *P* ≤ 0.05 from the two-way ANOVA analysis (Table S5). Gene's in bold have one or more phenotypes with a statistically different response to exogenous allyl treatment in comparison to Col-0. See Table 1 for T-DNA insertion lines details.

Using per seedling biomass, we utilized ANOVA based-tests to compare the exogenous allyl GSL response of the different T-DNA lines to WT Col-0 and showed that insertions in a number of genes abolished the Col-0 biomass and/or GSL response to exogenous allyl GSL treatment (Figure 6 and Table S5). For plant biomass, seven of the 13 candidate genes showed a significant interaction with exogenous allyl GSL treatment (*P* < 0.05) and three were suggestive of an interaction (*P* < 0.10). In addition, the T-DNA lines for eight of the genes also showed altered responses of endogenous GSL accumulation to exogenous allyl GSL in comparison to the WT Col-0. Interestingly, all of the T-DNA insertions that abolished the biomass response to exogenous allyl GSL also displayed an altered oxidation status of the 4C GSL away from the 4MTB and toward the 4MSB following exogenous GSL application (Figure 6 and Table S5). Thus, we can validate our ability to utilize the GWA mapping approach to identify genes that modulate the response to exogenous allyl GSL.

We also tested the ability of the T-DNA lines to alter the uptake and accumulation of the exogenous allyl GSL to test if the mutant effects were due to alterations in the metabolism of the exogenous allyl GSL rather than potential signaling effects. This is facilitated by the fact that the Col-0 background for the mutants has no functional AOP2 gene and as such cannot make nor convert the allyl GSL. As such, all measured allyl GSL in these samples had to come from the exogenous application. The level of accumulation of exogenous allyl GSL among the T-DNA lines was statistically identical to WT Col-0, except for AT4G16780 (ATHB2), which accumulated approximately 50% more allyl GSL in the leaves. Interestingly, this line was non-responsive to the exogenous GSL for both plant biomass and other aliphatic GSL traits (Figures 6A,B). This suggests that this increased uptake of allyl GSL was not causing hyper-responsiveness affecting plant biomass and endogenous GSL accumulation within this genotype. Thus, ATHB2 appears to modulate both the accumulation of exogenous GSL and the plant biomass to this exogenous GSL (Figure 6C). Further, these results show that the other genotypes all accumulated the exogenous allyl GSL to a level identical to WT Col-0 and any differences are likely from variation in other mechanisms. Thus, we can use GWA to find genes affecting the accumulation of exogenous allyl GSL and the response to this GSL.

## Discussion

A key limitation in modern biology is the ability to rapidly identify the genes underlying newly identified complex phenotypes/traits. GWAS are a promising route for dissecting natural variation by associating phenotypes with genotypes at a genome wide level. These studies exploit the nonrandom coinheritance of genetic variants (linkage disequilibrium) to simultaneously assay hundreds of thousands of markers for an association with any given trait. In contrast to the traditional use of structured mapping populations derived from two parent genomes, GWAS allow a wide sampling of the genotypes present within a species, increasing allelic variants per locus which allow potentially identify a greater proportion of the variable loci contributing to polygenic traits. In the present study, we used GWA mapping to begin to identify genes underlying the newly described trait wherein Arabidopsis modulates its plant biomass in response to a plant secondary metabolite. For that, we fed with exogenous allyl GSL a natural population of 96 Arabidopsis accessions. This population was chosen to minimize population structure and maximize statistical power for GWAS mapping with a moderate population size (Nordborg et al., 2002, 2005; Kim et al., 2007; Zhao et al., 2007; Atwell et al., 2010; Chan et al., 2010a,b, 2011). The application of exogenous allyl GSL caused changes in plant biomass and accumulation of defense metabolites identifying a wide range of genetic diversity across accessions varying between strong positive responses to strong negative responses. Utilizing natural variation of plant biomass, total amount of long-chain GSLs, total short-chain GSLs, total aliphatic GSLs, total indolic GSLs, and the sum of all GSL, we were able to identify genes within Arabidopsis that may control the biomass or GSL accumulation responses to exogenous allyl GSL. From all of the significantly associated genes with each trait, a small percentage was coincident between both conditions (control and allyl treatment; Tables S2, S3). Thus, the majority of identified GWA candidate genes are conditional upon the presence or absence of exogenous allyl GSL application.

A limiting factor for the utility of GWAS has been the preponderance of false-positive and false-negative associations which makes the accurate prediction of biologically valid genotype-phenotype associations very difficult. Integrating GWAS mapping results with additional forms of genome-scale data, such as transcript profiling or proteomics datasets has also been proposed to strengthen support for detected gene-trait associations and reduce the incidence of false-positive associations (Hawkins et al., 2010). We used a set of filters to reduce the number of candidate genes influencing plant biomass under exogenous GSL. First we removed the genes that were candidates in both conditions (control and allyl treatment). Then, the remaining genes were filtered based on their fold-change transcript accumulation is responsive to exogenous allyl GSL application (Burow et al., 2015). We selected 13 genes to test if T-DNA mutants of these genes altered this response (Table 1). Mutants in eight of these genes lead to a diminished or abolished response to the exogenous allyl GSL treatment (Figure 6A). In addition to altering biomass, these T-DNA lines also displayed an altered oxidation status of the 4C GSL (4MSB/4MTB ratio) following exogenous GSL application in comparison to the WT Col-0 (Figure 6B). Negative correlation between plant biomass response to exogenous allyl GSL and the ratio of methylsulfinylalkyl/methylthioalkyl GSL was reported before in Arabidopsis (Francisco et al., 2016). Of these eight genes, three had at least two mutant T-DNA alleles giving them stronger support for being true causal loci. It should be noted however, that all genes have at least two significant SNPs linked to the traits in question showing that there are two or more alleles within the natural accessions also linked to causing the trait variation. As was observed across the 96 Arabidopsis accessions, the mutants typically affected both the biomass and GSL responses, with only one mutant affecting only one or the other.

### Potential mechanisms of allyl response

These newly identified genes began to develop a crude model of how allyl GSL may influence biomass. Two of the genes, *HB4* (At2g44910) and *HB2* (At4g16780), are both homeodomain-leucine zipper II transcription factors that are important for controlling Arabidopsis development (Bou-Torrent et al., 2012; Nomoto et al., 2012; Carabelli et al., 2013; Turchi et al., 2013). *HB2* is also linked to altered auxin regulation suggesting that there may be a link between allyl GSL responses and auxin (Bou-Torrent et al., 2012; Nomoto et al., 2012; Carabelli et al., 2013; Turchi et al., 2013). Supporting this is the observation that another gene influencing the response to allyl GSL is At1g05680, *UGT74E2*, has been shown to alter in planta indole-3-acetic acid metabolism (Grubb et al., 2004, 2014). Further, auxin response networks are highly polymorphic within Arabidopsis (Delker et al., 2010). Interestingly, raphanusanin, a GSL specifically produced by *Raphanus sativa*, controls hypocotyl bending in response to light by affecting the TIR1 auxin receptor to modulate auxin signaling (Hasegawa et al., 2000; Yamada et al., 2003). Thus, it is possible that there are overlaps in how these two structurally unrelated GSL compounds, allyl, and raphanusanin, may affect plant growth in different lineages.

The remaining GWA candidate genes that we were able to validate as altering responses to allyl GSL are largely unstudied, including an F-box (At3g03040), a putative sphingolipid metabolism gene (At3g24460), a late embryogenesis abundant protein (At3g17520) and two proteins of unknown function At2g45360 (DUF1230) and At5g67370 (DUF1442). Thus, it is likely that these newly identified genes will allow us to identify new mechanisms controlling Arabidopsis plant biomass regulation. Extensive future studies will be required to map out how the defense metabolite allyl GSL can modulate various plant processes. Together, this shows that it is possible to use GWA mapping in plants to begin to identify genes controlling previously unknown traits, such as the ability to respond to endogenous secondary metabolites.

A key future step is to identify how Arabidopsis can detect allyl GSL and convey this information to the regulatory processes that control biomass and defense metabolism. Once these processes are understood, it will be possible to disconnect the potential feedback loop involving allyl GSL and formally test if the dynamic regulatory system becomes destabilized as predicted by theory.

## Author contributions

MF, MB, and DK conceived and designed the experiments. MF, HC, BL, CL, and RK conducted the plant work. MF, BJ, JC, and DK did the statistical analyses. MF and DK interpreted the data and wrote the paper.

## Funding

This work was funded by a Marie Curie International Outgoing Fellowship within the 7th European Community Framework Programme (PIOF-GA-2010-275286) to MF, the Spanish Ministry of Economy and Competitiveness through a ‘Juan de la Cierva’ program (IJCI-2014-19653) to MF, the NSF DBI grant 0820580 to DK, the NSF MCB grant 1330337 to DK, the USDA National Institute of Food and Agriculture, Hatch project number CA-D-PLS-7033-H to DK and by the Danish National Research Foundation (DNRF99) grant to DK and MB.

### Conflict of interest statement

The authors declare that the research was conducted in the absence of any commercial or financial relationships that could be construed as a potential conflict of interest.
